# Exploding the necroptotic bubble

**DOI:** 10.15698/cst2017.11.112

**Published:** 2017-11-07

**Authors:** Liat Edry-Botzer, Motti Gerlic

**Affiliations:** 1 Department of Clinical Microbiology and Immunology, Sackler Faculty of Medicine, Tel Aviv University, Tel Aviv 69978, Israel.

**Keywords:** necroptosis, phosphatidylserine, cell death, extracellular vesicles, apoptosis, annexin V, phagocytosis, necroptotic bodies, MLKL, RIPK3, RIPK1

## Abstract

The apoptotic death of cells is accompanied by the exposure of “eat-me” signals that serve to prevent necrotic degradation of apoptotic cells, and thereby prevent inflammation, promote resolution of immune responses, and stimulate tissue repair. These “eat-me” signals include the exposure of phosphatidylserine (PS) on the outer plasma membrane during the early stages of apoptosis as well as on the surface of apoptotic bodies, plasma membrane vesicles that are shed during the later stages of cell death. In our recent publication (PLoS Biol. 15(6):e2002711), we describe similar ‘eat-me’ and ‘find-me’ signals present during necroptosis, challenging some of our common assumptions about regulated forms of lytic death.

Several new types of regulated cell death have emerged from analysis of necrotic forms of cell death; one example is necroptosis, a RIPK3-MLKL-dependent and caspase-independent form of cell death. Although the initiation steps of necroptosis share some similarities with apoptosis, e.g. death receptors (TNFR1, CD95), necroptosis activation pathways typically feature inhibition of caspase-8 activity. As our knowledge of the molecular mechanisms of necroptosis increase, methods to help distinguish apoptosis and necroptosis are now clearer, for example caspase-8 activity for apoptosis and phosphorylation of MLKL for necroptosis. However, increasing complexity in these signaling cascades has been reported, and demonstrates considerable interplay including inflammasome activation and caspase activity downstream of MLKL phosphorylation. Therefore, there is a growing need for a re-evaluation of current assumptions about apoptosis and non-apoptotic pathways. This will require novel methods and more comprehensive analysis as a routine approach to distinguish the different cell death pathways.

We tested one of these methods, Annexin V staining, that is commonly used as a marker of apoptosis. This method relies on the detection of externalized PS, commonly seen in apoptotic cells but assumed to be absent in regulated lytic forms of cell death. Using several cell lines, we showed that PS is externalized to the outer plasma membrane of necroptotic cells prior to the loss of plasma membrane integrity. We demonstrate that phosphorylation of MLKL, a required step of necroptosis induction, plays an important part in PS exposure because inhibition of MLKL phosphorylation (using the specific kinase inhibitors for RIPK1 or RIPK3) blocked the exposure of PS. Furthermore, inhibition of pMLKL translocation to the membrane using the specific inhibitor necrosulfonamide (NSA) blocked PS exposure. Thus, apoptosis and necroptosis share more similarity in plasma membrane remodelling then previously assumed. The mechanisms leading to PS exposure in both forms of cell death remain to be fully defined, however, it is known that PS exposure during apoptosis is caspase-dependent and not MLKL-dependent. In addition, opposite to apoptotic cells, necroptotic cells with externalized PS were still not committed to die as addition of NSA to this cell population (to inhibit pMLKL membrane translocation) also rescues cell death.

PS externalization to the outer plasma membrane during apoptosis is known to be required for the recognition and engulfment of dying cells (‘eat-me’ signal), and to modulate the immune response. Thus, we decided to test if a similar pathway may be engaged in our PS-exposed necroptotic cells. Using four different phagocytosis models, we compared the ability of macrophages to phagocytose PS-exposed necroptotic cells *ex vivo *and* in vivo*. We demonstrate that necroptotic cells were phagocytosed more efficiently than viable cells but less efficiently than apoptotic cells. A change in the secretion of pro- and anti- inflammatory cytokines and chemokines was also evident following phagocytosis of apoptotic or necroptotic cells. Using a peritoneal inflammation model, we could show that necroptotic cells with externalized PS were highly phagocytosed in comparison to live cells but less efficiently than apoptotic cells. Finally, we determined that this phagocytosis is not due to any extrinsic effects as live cells were outcompeted by PS-exposed necroptotic cells in a competitive phagocytosis assay *in vivo*. These experiments not only confirmed that PS is exposed during necroptosis, but also reveals the potential of this signal to act as an ‘eat-me’ signal to alter the immune system.

Another feature of cell death that was assumed to differentiate apoptosis from inflammatory lytic forms of cell deaths was the generation of ‘apoptotic bodies’. These extracellular vesicles (EVs), which are between 0.5-2 μm, contain nucleic acids, proteins and organelles, which if release to the surrounding environment may serve as Damage Associated Molecular Patterns (DAMPs). Thus, apoptotic bodies limit the exposure of DAMPs to the surrounding extracellular space, and thus, are suggested to act as a specific alert system or ‘find-me’ signals to limit the inflammatory response. In this regard, we found that necroptotic cells also shed EVs prior to loss of cell viability or membrane integrity (**Figure 1**). These EVs, which we dubbed ‘necroptotic bodies’ were similar to apoptotic bodies in several aspects: they exposed PS to their outer membrane while some of them also contain proteins, including pMLKL. In contrast to ‘apoptotic bodies’, ‘necroptotic bodies’ did not appear to contain DNA. Furthermore, ‘necroptotic bodies’ (~0.2-0.8 μm) were smaller in size then ‘apoptotic bodies’ as determined by flow cytometry, transmission electron microscopy, size exclusion and NanoSight. Simultaneously, two other groups (led by Prof. Green and Prof. Wallach), describe that this EVs formation is dependent on pMLKL interaction with the ESCRTII machinery.

**Figure 1:**
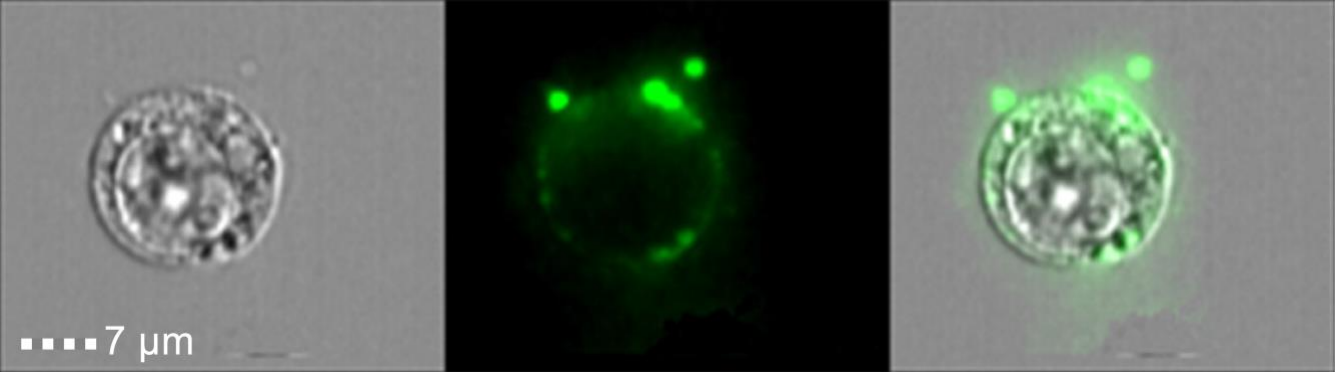
FIGURE 1: Phosphatidylserine exposure on necroptotic cells and ‘necroptotic bodies’ prior to outer plasma membrane permeabilization. U937 cells were stimulated for necroptosis by addition of TNFα (20 ng/ml), SMAC mimetic (20 μM) and zVAD (10 μg/ml). Four hours after stimulation cells were triple stained with Annexin-V-FITC (Green), Zombie (Purple) and PI (Red), and then analyzed by imagestream flow cytometry.

It is yet to be confirmed if ‘necroptotic bodies’ can be phagocytosed and/or can promote cell-cell communication. These necroptotic bodies may act as an alarm signal for surrounding cells, and thereby modulate the inflammatory response. Kinetic analysis of necroptosis using several different dyes revealed a delay in permeabilization of the outer membrane and the nuclear membrane, indicating that these processes are also regulated. Thus, we speculate that we have three waves of immunomodulatory processes that occur during necroptosis: the first (**Figure 2B**), generation of DAMPs trapped inside EVs (e.g. proteins including proteases and cytokines); the second (**Figure 2C**), the release of free cytoplasmic DAMPs; and the third (**Figure 2D**), the release of nuclear- and organelle-associated DAMPs (e.g. proteins, mitochondrial DNA and genomic DNA).

**Figure 2:**
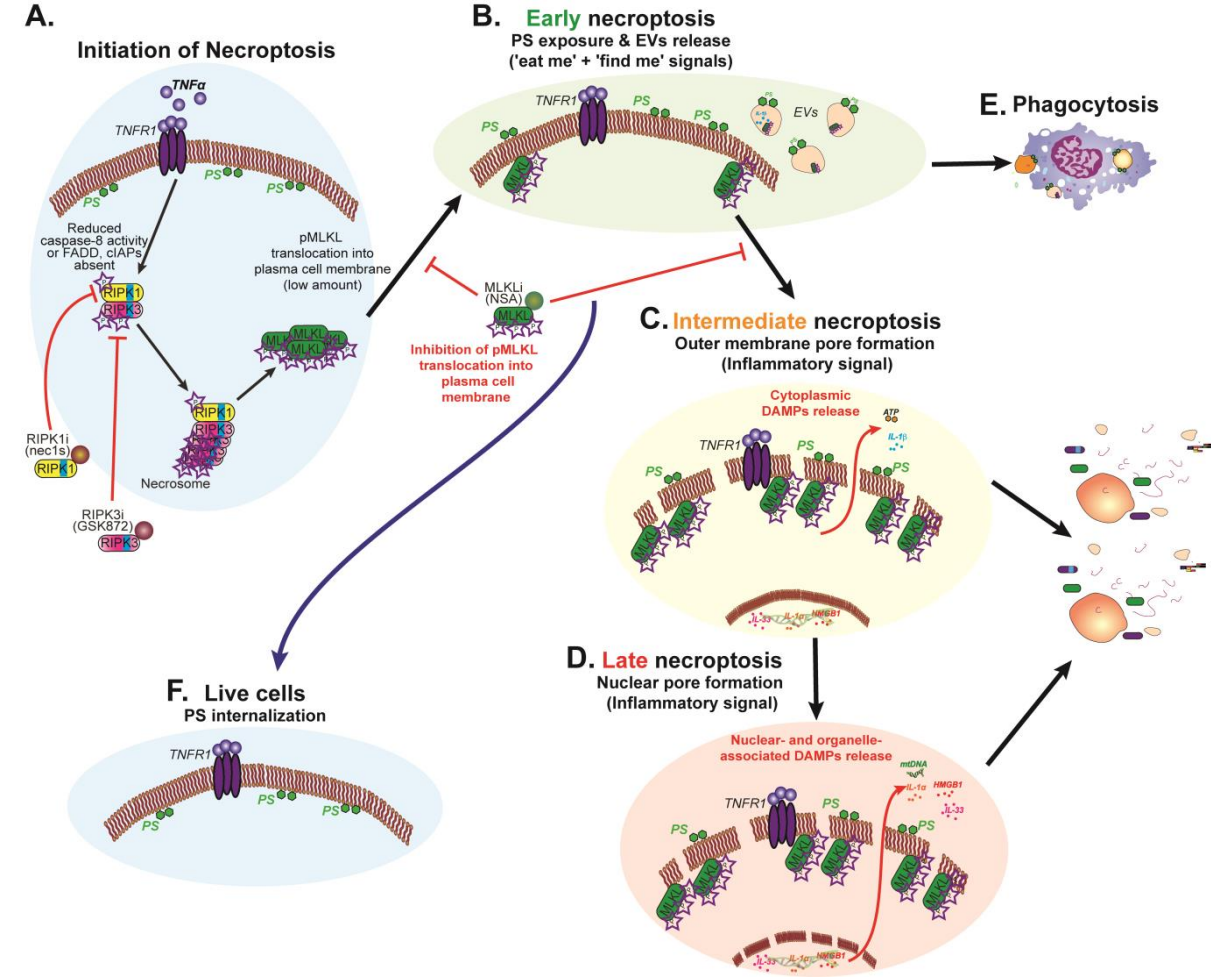
FIGURE 2: The three waves of immunomodulatory processes during necroptosis. Schematic summary and modelling of our results showing the three immunomodulatory waves during necroptosis. **(A)** Initiation of necroptotic signal transduction results in MLKL phosphorylation, triggering the first immunomodulatory signal **(B)** as phosphatidylserine (PS) is exposed on the outer membrane and ‘necroptotic bodies’ are released. **(C)** A second wave occurs when outer membrane integrity is compromised due to MLKL pore formation resulting in cytoplasmic DAMPs release. **(D)** The last wave of necroptotic immunomodulatory signal occurs when nuclear and organelle membrane integrity are lost leading to release of their DAMPs. **(E)** Phagocytosis of cells externalizing PS or ‘necroptotic bodies’ containing immunomodulatory intracellular factors. **(F)** MLKL inhibition can result in rescue of necroptotic cells in the early stages of necroptosis.

In summary, our findings that necroptotic cells externalize PS to drive recognition and phagocytosis, and to modulate the inflammatory response, provides a new insight into the specific ‘find-me’ and ‘eat-me’ signals generated during necroptosis, and change our understanding of this non-apoptotic form of cell death. These studies, together with two other studies (led by Prof. Green and Prof. Wallach) reveal surprising similarities between apoptosis and necroptosis and suggest that, as our knowledge of regulated forms of necrosis expand, additional assumptions that separate different forms of cell death will need to be reassessed.

